# Insulin-Degrading Enzyme: Paradoxes and Possibilities

**DOI:** 10.3390/cells10092445

**Published:** 2021-09-16

**Authors:** Malcolm A. Leissring

**Affiliations:** Institute for Memory Impairments and Neurological Disorders, University of California, Irvine (UCI MIND), Irvine, CA 92697, USA; m.leissring@uci.edu

**Keywords:** diabetes mellitus, insulin, insulin-degrading enzyme, proteolysis, protease inhibitors

## Abstract

More than seven decades have passed since the discovery of a proteolytic activity within crude tissue extracts that would become known as insulin-degrading enzyme (IDE). Certainly much has been learned about this atypical zinc-metallopeptidase; at the same time, however, many quite fundamental gaps in our understanding remain. Herein, I outline what I consider to be among the most critical unresolved questions within the field, many presenting as intriguing paradoxes. For instance, where does IDE, a predominantly cytosolic protein with no signal peptide or clearly identified secretion mechanism, interact with insulin and other extracellular substrates? Where precisely is IDE localized within the cell, and what are its functional roles in these compartments? How does IDE, a bowl-shaped protein that completely encapsulates its substrates, manage to avoid getting “clogged” and thus rendered inactive virtually immediately? Although these paradoxes are by definition unresolved, I offer herein my personal insights and informed speculations based on two decades working on the biology and pharmacology of IDE and suggest specific experimental strategies for addressing these conundrums. I also offer what I believe to be especially fruitful avenues for investigation made possible by the development of new technologies and IDE-specific reagents. It is my hope that these thoughts will contribute to continued progress elucidating the physiology and pathophysiology of this important peptidase.

## 1. Introduction

Biology is a curious mixture of science and art. On the one hand, X-ray crystallography can reveal the 3-dimensional structure of a particular protein in exquisite detail, right down to the atomic scale—reductionistic science par excellence. On the other hand, divining the functional role(s) of that protein more typically involves inference, intuition, and even guesswork—more like detective work than science—and decades can pass before complete elucidation of the protein’s physiological function(s). Such is the case for insulin-degrading enzyme (IDE; a.k.a. insulinase, insulysin; EC 3.4.24.56), an atypical zinc-metallopeptidase first described in 1949 [1,2], for which the crystal structure was obtained in 2006 [3,4]. In this commentary, I outline and discuss several outstanding questions about the biology and enzymology of IDE that warrant further elucidation, many of which take the form of intriguing paradoxes. By bringing these puzzling aspects into greater relief, I hope to identify lacunae in our understanding of the biology of IDE into which future research might shed new light.

## 2. Is IDE the Insulin-Degrading Enzyme?

IDE was first identified by I. Arthur Mirsky and colleagues by searching for a proteolytic activity capable of degrading insulin within crude extracts from tissues such as liver [1,2]. The designation of IDE as a peptidase of insulin specifically—rather than any other substrate, such as glucagon, amylin or amyloid β-protein (Aβ)—has had the effect of coloring our conception of its functional role ever since. Having been designated “the insulin-degrading enzyme,” it was natural to assume that IDE mediates insulin clearance in vivo. Subsequent research would show that IDE is ubiquitously expressed in both insulin-sensitive and -nonsensitive cells, where it is located primarily in the cytosol [5], basic facts in stark conflict with an exclusive role in insulin clearance, and yet this assumption held strong. Seventy-two years on; however, despite a few early animal studies reported findings consistent with this basic idea [6,7,8,9], there is now a growing body of evidence that casts doubt on this long-held assumption—or, at a minimum, offers compelling reasons to conclude that IDE mediates several other processes besides insulin clearance. The conflicting evidence was recently extensively reviewed [5,10], but the more glaring inconsistencies include a study from Eli Lilly reporting that a potent and selective IDE inhibitor they developed had no effect on insulin action in vivo as measured by euglycemic clamping (despite improving glucose tolerance) and, moreover, no effect on degradation of exogenous insulin in cultured cells [11]. In addition, genetic deletion of IDE in liver, the principal site of insulin clearance, failed to slow the clearance of circulating insulin [12], as IDE’s name would suggest; instead, rather paradoxically, overexpression of IDE in liver was found to reverse a diabetic phenotype induced by a high-fat diet [13]. Finally, the discovery that IDE appears to play a key role in—of all things—insulin *secretion* [14,15] is yet another beguiling rebuttal to long-held assumptions about IDE’s physiological function. As discussed more extensively in a recent review [5], these and many other observations suggest that Mirsky’s original idea that pharmacological inhibition of IDE could be used to treat type 2 diabetes (by slowing insulin clearance [16,17])—though certainly worthy of investigation—may need to be reconsidered (or at least refined) in the light of what we have learned about IDE in the intervening decades.

Thus, in my view, the name “insulin-degrading enzyme” has led the field astray. It is probably too late to change it, but a name lacking the word “insulin” would certainly help temper long-unquestioned assumptions about its function (perhaps “mesopeptidase” to distinguish it from exopeptidases, oligopeptidases, and proteases). At the very least, those of us in the field, and especially those entering it, ought to be advised to appreciate that IDE’s functions extend beyond insulin degradation—and, indeed, may perhaps have surprisingly little to do with it in vivo.

## 3. Where Is IDE?

Another discomfiting reality for the IDE field is persistent uncertainty about its subcellular localization. The vast majority of IDE is unquestionably present in the cytosol; IDE harbors no signal peptide [18] and is not secreted via the classical protein secretion pathway [19,20,21,22]. Consistent with a cytosolic localization, the peptidase appears to have no posttranslational modifications apart from acetylation of the N-terminus [23]. There is also definitive evidence for the presence of IDE within mitochondria. Alternative translation initiation of the *Ide* mRNA results in the addition of a 41-amino acid mitochondrial targeting sequence [24]. Expression of the mitochondrial isoform is limited, however, by the fact that the upstream initiation codon does not contain a strong Kozak consensus sequence [24,25]. Seemingly quite convincing evidence suggests that IDE is targeted to peroxisomes, as well [26]. The C-terminus of IDE ends in AKL, a tripeptide consistent with a type 1 peroxysomal targeting sequence (PTS1). More recently, however, the entirety of the PTS1 was shown to consist of 12 C-terminal amino acids [27], not just 3, and modern bioinformatic programs, such as *The PTS1 Predictor* [28], conclude that IDE is unlikely to be targeted to peroxisomes. Given this, there seems sufficient reason to re-examine whether this signal is a valid PTS1 or not. Finally, IDE was reported to be localized within endosomes [29,30,31,32,33] as well as associated with the plasma membrane [34,35,36,37,38]. However, much of the underlying evidence is decades old and frankly of mixed quality; moreover, some well-controlled studies have found no evidence for localization within either compartment, nor within lysosomes (e.g., [26]). Some of these contradictory findings may be explained if the subcellular localization of IDE is cell-type specific, but the possibility of contamination by mitochondrial (or peroxisomal) IDE even in the most carefully performed subcellular fractionation studies cannot be entirely excluded.

In a similar vein, the idea that IDE is present extracellularly seems well established, but precisely how IDE might be trafficked to the extracellular space remains obscure. For example, IDE was identified as the major protease responsible for degrading Aβ, a secreted peptide, in the conditioned medium of various cell types [39,40,41,42]. Moreover, genetic deletion of IDE increases [6,43,44,45] and overexpression decreases [46] levels of cerebral Aβ in vivo, consistent with the idea that IDE is present within the extracellular interstitial fluid in the brain (or one or more topographically contiguous subcellular compartments). Some groups have reported that IDE might be secreted from different cell types by various non-conventional export pathways [20,21,47], while other groups, such as Eli Lilly, simply found no evidence for extracellular IDE in cultured cells [11].

Could the “secretion” of IDE—particularly from cultured cells—represent non-specific spillage and/or basal turnover of cytosolic contents? Consistent with this view, Hersh and colleagues conducted a careful study that found that IDE secretion from multiple cultured cell lines tracked perfectly with generic markers of cytosolic proteins, such as lactose dehydrogenase (LDH) [19]. Similarly, in an unpublished cell-based compound screening campaign of ~1.2 million drug-like molecules, which monitored IDE activity and LDH release in parallel, my group found that all compounds that increased extracellular IDE activity also increased LDH release (M. L. unpublished observations).

In my opinion, the field needs to revisit the questions of where in the cell IDE is located, whether it is secreted and, if so, precisely how. Rather than relying exclusively on published reports, most several decades old and some using less-than-ideal methodologies, we should make full use of the powerful new tools now available, such as IDE-null cells [6,8], multiple monoclonal antibodies [48], and IDE-specific molecular probes [9,49,50,51,52], which should be deployed in tandem with modern labeling and imaging techniques. As for the secretion of IDE, I believe more in vivo studies are needed to verify whether IDE is (ordinarily) present extracellularly and, if so, to what extent. Current evidence for IDE within plasma [20,21,53,54,55] is largely indirect, often relying upon antibody capture approaches coupled with activity assays (which have their own problems; see below). Using physiological levels of radiolabeled peptides, my own group found vanishingly little Aβ- or insulin-degrading activity in neat, EDTA-free plasma, and we also found an underwhelming amount of Aβ-degrading activity sensitive to IDE-specific inhibitors in CSF (M. L. unpublished observations). It may be that IDE is secreted but nonetheless relatively inactive in plasma and CSF, due to specific or non-specific endogenous inhibitors, in which case immunoprecipitation experiments would be particularly appropriate. These topics are of paramount significance to the field, particularly to many long-held assumptions within it, and thus are deserving of being intensively investigated anew.

## 4. Enzymological Paradoxes and Problems

Numerous X-ray crystallographic and cryo-electron microscopic studies of IDE have now been conducted [4,56,57,58], revealing that IDE exists as a homodimer, with each unit being comprised of two bowl-shaped halves connected by a linker, resembling a clamshell. Each monomer can adopt “open” and “closed” conformations, completely encapsulating a large internal chamber when “closed” [4,58]. Notably, the active site is bipartite, with portions present in both the N- and C-terminal halves, becoming fully functional only in the “closed” conformation, and accessible exclusively from within the internal chamber [4,56,59]. This distinctive structure explains several previously puzzling properties of IDE. For instance, because substrates must fit entirely within the internal chamber to be processed, this explains why IDE can only degrade peptides while being unable to process proteins. Moreover, because substrates make extensive contacts throughout the internal chamber, including at multiple sites distal to the active site, this explains IDE’s otherwise baffling substrate specificity, such as how IDE avidly degrades IGF II, for example, but only slowly degrades similarly sized, related substrates such as IGF I [60,61,62]. The extensive and variable contacts made between different substrates and IDE also help explain why small-molecule modulators of IDE frequently show striking substrate-specific differences in potency; indeed, compounds that activate the degradation of some substrates can actually inhibit the degradation of others [49,63]. Most recently, this peculiar property has been quite usefully exploited to develop insulin-specific inhibitors of IDE [50].

At the same time, IDE’s peculiar structure also raises several intriguing paradoxes. For example, what prevents IDE from becoming quickly and hopelessly “clogged up” by any of the myriad peptides and other intermediate-sized molecules in its vicinity? Just considering its known substrates, IDE has been shown to form stable, irreversible complexes with both Aβ [64,65] and α-synuclein [66,67]. Even highly purified, extensively washed, recombinant IDE used for crystallization experiments has invariably been found to contain non-specific peptides within its internal chamber [56]. Some have proposed that this feature of IDE may reflect a “dead-end chaperone” function, possibly serving to sequester amyloidogenic peptides [15,65,67]. Perhaps. However, if this occurs for some peptides, it likely occurs for many more. My question is: what keeps IDE from getting clogged? I believe this is a genuine conundrum deserving, at a minimum, of further research and, ultimately, of a mechanistic explanation.

Another, potentially related question exists: if IDE exists predominantly in the “closed” conformation, as most crystal structures show and other evidence suggests [56,57], what triggers the transition to the “open” conformation? Inter-subunit cooperativity may provide part of the explanation, with the action of one subunit affecting the other allosterically [68], and recent cryo-electron micrographic studies seem consistent with this idea [58]. Nevertheless, it is intriguing to ask: what if anything causes IDE to open? Can substrates participate in this process?

There is also the curious fact that there seems to be an uncannily snug fit between insulin and IDE’s internal chamber [4,58,69]—could there be a functional significance to this or is it mere coincidence? Here, I offer a speculative but intriguing possibility: perhaps the binding of insulin to IDE could serve as a signal of sorts—i.e., an indicator of the presence of insulin—transmitted to other cellular components in turn via a conformational change, for example. Such a signaling event might even occur in the cytosol, given the considerable (and surprising) literature suggesting that insulin can in fact reach the cytosol and even the nucleus [70,71,72,73,74,75]. In this admittedly highly speculative scenario, hydrolysis of insulin by IDE would constitute a mechanism for resetting the signaling mechanism to the initial state.

In concluding this section on enzymological properties of IDE, I would like to make a sincere appeal to those in the field: please stop using short fluorogenic peptide substrates to monitor IDE activity. These substrates are ill-suited for multiple reasons. First, they are prone to degradation by numerous off-target proteases and to that extent are ill-suited for applications with biological materials (unless IDE-specific inhibitors are used to distinguish IDE activity from non-IDE activity). Second, the processing of such short peptides by IDE differs in significant ways from the processing of larger substrates. By virtue of their larger size, physiological IDE substrates such as insulin and amylin interact with a distal exosite, which appears to be critical for substrate recognition and processing [76]. Shorter substrates, by contrast, do not [77]. As a consequence, when used for compound screening or in vitro activity assays, short fluorogenic peptides frequently lead to the identification of “activators” that prove to be nothing of the kind when tested against more physiological substrates [63,78]. In my considered opinion, these compounds “activate” IDE for a trivial reason: they occupy a portion of the internal chamber of IDE, therefore reducing the remaining volume available to the short fluorogenic peptide and in this way, increasing the likelihood of making fruitful contact with the active site of IDE. While it may one day be possible to identify bona fide IDE activators, the search needs to be conducted with physiological substrates, not fluorogenic peptides—and so far my own laboratory’s efforts to identify activators using this approach, including testing ~2 million compounds in multiple compound screening campaigns, were unsuccessful (M. L. unpublished observations). Precisely to overcome the limitations of short fluorogenic substrates, and to facilitate these screens, my laboratory expended considerable efforts developing several well-characterized and highly quantitative assays for multiple physiological substrates of IDE [79,80,81]. Given the availability of these considerably more reliable IDE activity assays, which use inexpensive reagents and can be readily implemented by any laboratory equipped with a fluorometer, there is, in my opinion, no justifiable reason to rely on short fluorogenic peptides when seeking to quantify IDE activity.

## 5. IDE Partners?

Does IDE primarily exist as a free-floating, self-sufficient, homodimeric protein, operating in isolation? This view does indeed seem supported by several facts, including the high activity of purified recombinant IDE, the fact that it is found predominantly within the soluble fraction of cells, while also being secreted. However, over the years, IDE has been suggested to interact with a variety of components, including the androgen receptor [82], the proteasome [83,84,85], certain membrane associated phosphoinositides [86], among others (reviewed in [5]). However, some of these interactions were merely postulated and others not confirmed by independent groups.

I feel strongly that the field would benefit significantly from a thorough search for potential partners of IDE, proteinaceous or otherwise. Protein partners and/or new substrates might be discovered via many possible methodologies, including in vitro transcription/translation approaches with immobilized IDE, co-immunoprecipitation experiments with anti-IDE monoclonal antibodies coupled with mass spectrometric analyses, and/or by approaches using reversible crosslinking agents. Biotinylated versions of IDE-selective probes of IDE, such as 6 bK [9] or even insulin, could also be exploited to search for both proteinaceous and non-proteinaceous interacting partners. In contrast to earlier decades, numerous, very powerful technologies are available to the modern researcher, and a thorough search IDE-interacting partners could very possibly lead to fruitful discoveries of great importance to the field.

## 6. Possibilities

Thanks to recent advances both within the IDE field and in biology generally, many exciting possibilities for future investigation are now available, which have—rather surprisingly—remained relatively unexplored. Some of these opportunities hold great promise for helping to resolve the longstanding paradoxes discussed above, while others may open new avenues of investigation and/or new therapeutic advances.

Many of the most exciting new possibilities hinge on the development of powerful pharmacological modulators of IDE. It needs to be emphasized that until 2010—more than 60 years after the discovery of IDE—there were literally no selective pharmacological inhibitors of IDE or any member of its unusual peptidase superfamily [87]. Since that time, numerous inhibitors were developed that are: (1) potent and selective for IDE [9,11,50,51,87,88,89]; (2) in vivo compatible [11,52,89]; (3) selective for extracellular IDE exclusively [52,90]; (4) inexpensive to synthesize [49,51]; and (5) substrate selective [88,91,92], with many compounds with multiple meritorious properties simultaneously. These IDE inhibitors represent a powerful yet essentially untapped resource for the field, which could be used to address numerous topics. In this regard, it is important to emphasize that both proteolytic and non-proteolytic functions were assigned to IDE [5,93]. Up to this point; however, most manipulations of IDE have been limited to genetic deletion or overexpression, which modifies not just the activity but also the protein itself. Studies conducted with pharmacological inhibitors, by contrast, would permit the proteolytic activity of IDE to be interrogated while leaving the protein itself unperturbed, which may yield fresh insights into IDE’s function. IDE inhibitors might also be useful for clarifying precisely where IDE exists within cells: in a simplistic example, if IDE exists in endosomes, then extracellular application of non cell-penetrant compounds (or those active extracellularly only [52]) should effectively block IDE activity within endosomes (and the extracellular space), while sparing IDE activity in other cellular compartments. Additionally, the newly developed inhibitors can be used to explore the therapeutic potential of IDE inhibition in domains besides diabetes, such as wound healing [94,95,96], as made possible by the invention of cost-effective peptidic IDE inhibitors [51]. Last but not least, the development of an insulin-specific IDE inhibitor by Liu and colleagues [50] is an extraordinary achievement that at long last permits us to explore IDE’s impact on insulin independent of the confounding effects of other IDE substrates that complement or counteract insulin action (e.g., amylin, glucagon). This very exciting new class of inhibitor has immediate potential for elucidating the exact relationship(s) between IDE and insulin signaling in cultured cells and ex vivo tissue culture and—perhaps after suitable modification—might eventually be used to help clarify IDE’s role(s) in insulin homeostasis in vivo.

The relatively recent development of genetically modified mouse lines permitting inducible and tissue-specific genetic deletion of IDE has helped significantly advance the field, while at the same time raising new questions about IDE’s physiological functions [10,12,13,14]. More studies of this kind seem warranted. In particular, longitudinal studies that follow the consequences of inducible deletion of IDE—triggered after development, either throughout the body or in specific tissues or cell types—would be especially illuminating, because they could help distinguish primary consequences of IDE deletion from secondary ones, which may include confounding compensatory changes [10].

The advent of powerful new gene-modifying technologies, such as CRISPR-Cas9 [97], also opens up fresh avenues for experimental investigation. The significance of the mitochondrial variant of IDE, for instance, could be investigated readily by inserting a point mutation at the upstream initiation codon that precludes translation of the mitochondrial targeting sequence (or conversely, by mutating the downstream initiation codon to preclude non-mitochondrial variants) [24]. The putative PTS1 signal at the C-terminus of IDE could be interrogated similarly. In addition to (or rather than) generating novel mouse lines, these exciting technologies could also be used to generate pluripotent stem cells, which could then be tested in cell culture after differentiation into different cell types. A complementary cell culture approach would be to generate immortalized cell lines lacking IDE, then express different variants of IDE—such as fluorescently tagged versions—in these IDE-null cells to investigate various topics.

## 7. Conclusions

In summary, I sought herein to illuminate a few of the more puzzling aspects surrounding the biology and pharmacology of IDE, as well as to point out a few relatively untapped possibilities for future research. I hope this might help in some small way to inspire previously unconsidered research directions, which in turn may help resolve some of the many paradoxes within the field. IDE is an important peptidase with strong links to several human diseases, so resolving these outstanding questions will expand our understanding of these disorders and, potentially, could lead to novel therapeutic approaches.
